# Considerations on the Relative Development and Functions of the Different Parts of the Nervous System in Man and Brute Animals; Leading Especially to a Comparison between the Powers of the Five Senses in the Same Beings

**Published:** 1820-01

**Authors:** 


### COMPARATIVE ANATOMY AND PHYSIOLOGY.

***Considerations on the relative Development and Functions of the different Parts of the Nervous System in Man and Brute Animals; leading especially to a Comparison between the Powers of the Five Senses in the same Beings.***

THE utility of comparative anatomy and physiology in the consideration of the structure and functions of man has become so generally acknowledged, that it is unnecessary to make any reflections, in order to impress a sense of it on the mind of medical enquirers, though but few of them have even a general knowledge of this subject, in consequence of the extensive researches that are requisite for its acquisition, through many details more or less uninteresting to them. Some considerations on those points only which are especially connected with the most important of the functions, avoiding details improper for the plan of a Medical Journal, appear to me to promise being of utility, as well as interesting, to those I address; and I propose to offer them, in the first instance, on the structure and functions of the nervous system in the class of vertebral animals.

Although we have but little precise knowledge of the functions of particular portions of the encephalon, I shall commence with some general, and a few particular, observations respecting the parts composing it; as several well-ascertained facts seem to present interesting matter for reflection.

The brain, the centre of the motive powers of animal life, is always contained in an osseous envelopment, and, by its assemblage with the face, constitutes the head of all animals. The relation in volume between the parts of the head just distinguished, is of the highest interest; because the osseous envelopment, or cranium, shows the volume of the brain, and consequently that of the organ of the intellec and voluntary power: whilst the face is the seat of three of the four most important senses of the inferior animals; and, as the nature of every animal depends in a great degree on the relative energy of its functions, and as the animal is in a manner directed and governed by those of his sensations which are the most powerful, according to Cuvier,* the volume of the face forms also an interesting subject for consideration.

Aristotle, Erasistratus, and Pliny considered that the intelligence of an animal existed absolutely in a direct ratio to the bulk of the cerebral mass; and therefore that man, as they supposed, possessing the largest brain of all animals, was also the most intelligent. But the erroneousness of this statement soon became evident; for it was found that the brain of an elephant is about twice the size of that of a man, weighing about seven pounds, according to Moulins; whilst the medium weight of that of a man is but about three pounds and a half, or four pounds. An opinion was then advanced, that the same proposition would remain correct, if applied in a relative manner to the bulk of the rest of the body; and this at first appeared to be well substantiated: but this is not universally the case, and therefore the proposition fails in its express and most important object. Many small birds have a brain much larger in proportion to their body than man: sparrows, chaffinches, goldfinches, and canary-birds, have it in the proportion of from the 32° to the 25°, and even some to the 14° of their body, according to the researches of Pozzi; whilst in man it is in the proportion of about one to forty. Observations on various other species of animals, seem also to show the incorrectness of this notion; though the bulk of the cranium, it should be remarked, is not in every instance a precise criterion for that of the brain. The *physeter macrocephalus* (Lin.) has, perhaps,

* *Leçons d'Anatomie Comparée*, t. ii. p. 3.

the most enormous head of all animals, relatively speaking with respect to the body of the animal; but his brain swims in a flood of oil, which, when concrete, forms spermaceti, so that it does not nearly fill the cranium. This is also the case with respect to the dolphin. In general, small animals have proportionally more brain than larger ones. An elephant, weighing five thousand pounds, has, according to Moulins, but about seven pounds of brain; a great part of his voluminous head being constituted by the spacious cavities serving as olfactory sinuses. An ox, of nine hundred pounds weight, has but from sixteen to twenty ounces of brain; a horse, of seven hundred pounds weight, twenty ounces, or somewhat more, which is only in the proportion of about one to five hundred; whilst in the ass, considered so stupid, it is as one to one hundred and fifty. In the beaver, on the other hand, apparently so intelligent, the proportion is as one to about two hundred and ninety or three hundred. In some carnivorous animals, as the cat, the proportion is from the one-hundredth to the one-hundred-and-fiftieth part of the body: in the dog and the wolf it varies from the last-mentioned number to the two hundred or two hundred and fiftieth part. In some frugivorous animals it becomes much greater; for it forms the two hundredth part in the hare, and the one hundred and fortieth in the rabbit. It is very considerable in rats and mice: in the former it is as one to about seventy, in the latter as one to fifty, with respect to the bulk of the body. Amongst the ape tribe the proportion is very great: a baboon, of the same size as a fox, has much more brain than this animal, according to WILLIS. The pygmy (*simia troglodytes*, LIN.) dissected by Dr. TYSON, an animal only twenty-six inches in height, had eleven ounces seven drachms of brain; which, as BUFFON remarked, is nearly equal in proportion to the body with that of man.

The foregoing proposition is, however, to a certain extent, valid; and it should be remarked, that the cranium of an European will contain, on an average, from four to six ounces more water than that of a negro, and that of men of all races about the same quantity more than that of women of the same tribe respectively.

Another proposition was then made, that the brain of man is most considerable in respect to the rest of the nervous system, comprising the ganglionic centres and their productions, as well as the spinal marrow and its nerves, and those of the brain properly speaking: and this has hitherto remained unrefuted. There is a difference, too, in the proportion of different parts of the encephalic mass that is deserving of notice: the anterior lobes of the cerebrum being much more considerable in man than in brute animals, and even in some races of men than in others; and this in a direct ratio to the excellence of their intellectual powers. It is on this basis that CAMPER raised his proposition of making the angle formed by a line drawn from the superciliary ridge to the alveolar processes of the upper jaw, or rather, as CUVIER advises, to the basis of the nasal foramina, and thence carrying the line through the external auditory foramen to the basis of the cranium, a standard for determining the degree of intellectual power. This angle is more obtuse in man than in any other animal. In the *European* this, the *facial angle*, is from 80° to 85°; in the *mongolic race*, about 75°; in the *negro*, 70°; *oran-outang*, but 65°; in the *sapaji* and *sagoini*, about 60°; in the *lamures*, of the varieties *mongoz* and *macoco*, 45°; in the *mandril*, 30°. But, with Cuvier's modification, the facial angle is only important with respect to the human species and the *quadrumanes*, because they have only very small frontal sinuses, which do not open the facial angle in a sensible manner, and because the bony part of the nose in them is chiefly below the perpendicular line: whilst some carnivorous, and a few ruminating, animals, especially the *elephant*, have such large frontal sinuses, that they throw the extremity of the perpendicular line too far forward in proportion to the *real bulk* of the brain. Thus, the parts of the brain forming the organ for the development of the mind, seem to be especially the anterior lobes of the cerebrum; whilst the posterior lobes, and the cerebellum, appear rather to be subservient to the vital, and, as they are commonly termed, the animal, functions. This proposition respecting the formation of the head will be found to be supported by observations made on Europeans. Men of great intellectual powers present almost universally a large, expanded, forehead, having a direction nearly perpendicular with respect to the face: whilst idiots, and especially the Cretins and Cagots of the Alps and Pyrenees, have a forehead approaching in shape to that of the negro, and even, in many instances, nearly similar to the oran-outang. These circumstances did not escape the observation of the Greeks: we find, in their figures of the divinities, and even in those of some men, the eminence and projection forwards of the forehead carried to an extent that was probably never witnessed in man.

The relative difference between the bulk of the brain and the rest of the nervous system, with respect to man and brute animals, was first clearly pointed out by SOEMMERING and EBEL; and, as the face is chiefly constituted by the development of some of the organs of the senses, this seemed to furnish a basis for determining in some degree the relation between the size of the brain and the rest of the nervous system above designated. This has been favoured by CUVIER, who advances as general propositions, that "man, of all animals, has, proportionally, the largest cranium and smallest face; and that animals deviate from these proportions as they become more stupid and ferocious." In the latter, the animal appetites and passions have the superiority in the economy;* and hence the difference between the Hercules and the Apollo of the Grecian statues. These propositions hold good with respect to individuals of the same race, as well as to different races of men; and may be confirmed by daily observation. The great and projecting mouth, with large and turned-up nostrils, and a low and retreating forehead, were seized by Hogarth to picture ugliness: they equally designate a superiority of the disposition to the brutish passions over the amiable qualities of the mind, in the economy of man. This separation of the cranium from the face should be made by a line drawn in a curved direction, so as to inclose with the surface of the cranium an oval space, from the occipital foramen to the root of the nose, so that the great axis of the line may be on a parallel with the lower part of the nasal bones. In the European, the area of the cranial cavity is nearly quadruple that of the face, not comprising the lower jaw. In the negro, supposing the area of the cranium to be the same, that of the face would be augmented about one-fifth; and in the Calmuck only about a tenth. In the sapaji, the area of the face is nearly half that of the cranium. In the mandril, and nearly all the carnivorous animals, except the *molossus* variety of dog, which has a short nose, they are nearly equal. In the ruminants and solipedes, all have the area of the face greater than that of the cranium. The line drawn in the manner directed forms, with the surface of the cranium, nearly a circle in the European; in the negro it is a more flat oval; in the carnivorous race of animals it is semi-elliptic, a little rounded above, the basis of which is nearly equal to the height.

The general disposition of the nervous system is nearly the same in all vertebral animals. The cerebrum, contained in the cranium, presents a mass much more firm than that of invertebral animals. It is always accompanied with a tubercle termed the cerebellum, from which two bands are detached, that pass through the occipital foramen, and then take the name of spinal marrow. These bands are generally found firmly united at the period of birth; but Zacchias, Manget, Grashuys, Hall, Malacarne, and Mohrenheim, relate instances of their being distinct in the human fœtus at that epoch. All the nerves leave the brain and spinal marrow in pairs, and are distributed over the body, not like the branches of a tree, but anastomosing so as to form plexuses, from which more filaments are sent off than

* Cuvier, *Leçons d'Anatomie Comparée*, t. ii. p. 3.

what are given to them. The spinal marrow is ordinarily larger than any of the nerves that are connected with it; and the encephalic mass surpasses in size the spinal marrow.

Although the development of the cerebral nervous system is conformable to certain general laws in the whole class of vertebral animals, yet there are some varieties in this respect worthy of notice.

The brain of fishes is an assemblage of small lobes, not constituting one mass, but forming a double congeries of tubercles united above. It does not entirely fill the cranium, and is always very small in proportion to the bulk of the body. The surface of the hemispheres of the brain is smooth. The cerebellum is relatively larger than in other animals. The whole encephalic mass is detached from its membranes, except the pia-mater, which is the only one of them immediately covering it. The space between the membranes is filled with a loose, thick, cellular substance, infiltrated by a gelatinous fluid in cartilaginous fishes, sanguineous in others, and oily in the carp and salmon. This cellular structure is covered by the dura-mater, which is adherent to the cranium, and does not form any folds.

The encephalon of reptiles resembles in general that of fishes. The cerebellum is, however, proportionally smaller, and the spinal marrow more slender.

This mass in birds is composed of six tubercles, very distinctly visible: two form the hemispheres; two, the thalami optici; one, the cerebellum; and the last, elongating itself, the spinal marrow. The pia-mater is prolonged into folds, which penetrate between the circumvolutions of the medullary substance, and two narrow bands pass into the ventricles. Above this is the arachnoid membrane, which assumes the place of the humid cellular substance of cold-blooded animals. It does not appear to penetrate into the ventricles, but forms sorts of bridges over the grooves between the convolutions of the brain; and it is continued in the same manner over the spinal marrow. The dura-mater forms several prolongations, which pass between the tubercles of the brain already described.

The anatomy of the brain of the inferior families of the class of vertebral animals, is a subject urgently demanding attention; especially in relation to the opinions of Carus,* and some other German anatomists,—that the brain is formed by a union of the ganglions of the nerves of sense, and by the central mass of the spinal marrow. The distinctness of different parts of the brain of birds corresponding to the terminations of the optic

* *Versuch einer Darstellung des Nervenystems, und insbesondere des Gehirns, &c* Leipzick, 1814.

nerves, is also acknowledged by TIEDMANN, (see his *Zoologie*, Heidelberg, 1808-1814,) since this was so clearly demonstrated by GALL. On these principles, each lateral division of the encephalon is constituted of three grand masses: 1, that formed by the development of the extremity of the organ of smelling; 2, that of the sense of sight; 3, that of the nerves of motion: an inferior mass is, that of the nerves of hearing. And some anatomists have supposed that they have discovered a particular formation and development of the osseous envelopment corresponding to them,—in, 1, the frontal bone; 2, the parietal bones; 3, the occipital bone; and 4, the temporal bones.*

The brain of the mammiferi does not differ in many apparently-important circumstances from that of man: the difference chiefly consists in the greater or less development of the hemispheres, which are thicker in man than in any other animal. In apes, they begin to become flattened at the sides, and to be more prolonged backwards. In the carnivorous brutes, the anterior lobes are very flattened, the middle ones much less projecting downwards than in man, and the posterior ones wholly wanting: the cerebellum is seen from above perfectly uncovered. WENZEL (*De Penitiora Structura Cerebri*) states, that the cerebellum is proportionally larger in some of them. This part of the encephalon is more simple in its appearance in the inferior families of the class of animals under consideration, than in man; but it is always composed of two lateral portions. In fish and reptiles, its surface is plain, and we see none of those divisions which, by a perpendicular incision, present in the higher orders the appearance termed the arbor vitæ. But, in birds, that appearance may be discerned, and the surfaces present somewhat of the grooved appearance so much more conspicuous in quadrupeds and man; in the carnivorous animals they are more clearly marked. Some of the mammiferi have a fine net-work of vessels, given off from the carotids as they enter the cavity of the cranium, formed at the basis of the brain, especially about the pituitary gland, that is wanting in man.

The most remarkable differences in the more interior parts of the brain, is that, as animals recede from man, the tubercula

* See *Ueber die Bedeutung der Schædeiknochen*, von L. OKEN, Jena, 1807; and *Die Zeugung*, Bamberg, 1805, by the same author. *Cephalogenesis, sive Capitis Ossei Structura, Formatio et Significatio per omnes Animalium, Classes, Genera, ac Ætates Digesta;* auctore J. B. SPIX; fol. Munich, 1815. *Epist. de Cur. Homin. Morb.* auctor J. P. FRANK, I. ii. *Cours d'Etudes Medicales*, par M. BURDIN, tome i. *Memoires de Zoologie et d'Anatomie comparée*, par M. DUMERIL; Paris, 1808. *Beytraege zur Vergleichenden Anatomie*, von J. F. MECKEL. *Versuch einer Darstellung des Nervensystems, und insbesondere des Gehirns, nach ihrer bedeutung entwickelung und Vollendung im thierischen Organismus*, von C. G. CARUS; Leipzick, 1814. *Philosophie Anatomique des Organes Respiratoires, sous le Rapport de la Determination et de l'Identité de leurs pieces Osseuses*, par G. SAINT-HILAIRE; Paris, 1819. And *Mémoire sur l'Osteose*, par M. BECLARD; Paris, 1819.

quadrigemina become proportionally larger:* or it may be better to sum up the differences at once, and say, that the proper characters of the brain of man and apes, consist in the existence of the posterior lobes, and the digital prolongations of the third ventricles dependant on the existence of those lobes; that of the carnivorous animals, in the smallness of the nates relatively to the testes; that of the frugivores, in the largeness of the nates, and the absence or superficiality of the convolutions; that of hoofed animals, in the bigness of the nates, joined to numerous deep convolutions; that of the cetaceæ, in its high or pyramidical shape, and its large size, and in the total absence of the olfactory nerves; the herbivores all have the nates larger than the testes; man and the quadrumanes have alone the olfactory nerves, properly speaking: the place of them is filled in the true quadrupedes by mamillary tubercles; and they are wanting in the cetaceæ.

All the parts of the brain of reptiles are smooth, and without circumvolutions. The optic nerves are placed behind the hemispheres, and are not covered by them. They each contain, as in birds, a ventricle which communicates with the third. We see, at the two extremities of these, the anterior and posterior commissures; but there is no soft commissure, and there are no tubercula quadrigemina. After marking the most important differences in the families of vertebral animals, it may be useful to give the proper character of each order:

1. The distinctive character of the brain of the mammiferi from that of other red-blooded animals, consists,—*a*, in the existence of the corpus callosum, the fornix, the cornua ammonis, and the pons Varolii; *b*, in the position of the tubercula quadrigemina on the aqueduct of Sylvius; *c*, in the absence of any ventricle in the thalami optici, and in the position of the thalami within the hemispheres; *d*, in the alternate lines of grey and white substance in the interior of the corpora striata.

2. The characteristics of the brain of birds are—*a*, the thin and radiated membrane which closes each anterior ventricle on the internal sides.

3. Those of the brain of reptiles are—the position of the thalami optici behind the hemispheres.

4. Of fish, in the tumors of the olfactory nerve, and in the tubercula being behind the cerebellum.

5. Those of the three last consist in,—*a*, the want of corpus callosum, fornix, and dependences; *b*, in the presence of more or less numerous tubercles situate between the corpora striata and the thalami optici; *c*, in the existence of ventricles in the thalami optici, and the disengagement of these from the

---

* Cuvier, *Leçons*, &c. t. ii. p. 153.

hemispheres; *d*, the absence of any tubercle between the lateral portions of the cerebellum, as well as of the pons Varolii.*

The most remarkable difference in the spinal marrow between the different classes of vertebral animals, is the greater or less distinctness of each of the portions of it corresponding to the bodies of the vertebræ. In man, this part of the cerebral system presents a continuous cord, almost equal in diameter, except that it is larger about the thorax than towards the loins: whilst, as we verge towards the invertebral animals, each of the portions alluded to becomes more distinct; until at last they form a chain of ganglions, united by slender nervous cords; and the distinctness of the influence of each portion on the system, is in a direct ratio with the geometrical distinctness above marked out. The spinal marrow is primitively found in all animals in distinct portions, according with each of the vertebræ; that is, in the form of a chain of glanglia. Now, Carus lays it down as a general principle, that the more perfectly an organ bears the character of its primitive form, the greater, relatively, are its powers in the economy; and, in conformity with that principle, it appears that, in the more perfect animals, when the spinal marrow has become formed into a sort of nervous cord, it is the upper ganglion developed into the encephalon, presenting the type of anterior formations in a strongly-marked degree, which possesses the superiority. In the lower orders of animals of the same class, the spinal marrow, preserving its primitive character, is the grand centre of nervous influence. Voluntary power, and its consequent muscular force, are in the latter, then, exchanged for a more perfect existence of the relative senses, and consequently of intelligence. The encephalon of the lower orders of the vertebral class of animals, (that is, those of cold blood,) it may here be proper to repeat, is more or less smooth and uniform, not presenting any of that lobulated formation so well developed in man.

The difference in the notions of Hippocrates, who regarded the brain in general as the organ of the mind; of Plato, who considered this to be situated in the spinal marrow; and of Aristotle, who placed it in the epigastrium;—notions that have been renewed in modern times,—seem to have arisen from the different views they took of the animal functions, and the different orders of animals from the consideration of which they drew their conclusions.

The general distribution of the cerebral nerves is not essenially different in any of the orders of vertebral animals. The varieties in this respect that seem worthy of notice will be de-

* CUVIER, *Leçons*, &c. t. ii. p. 171.

signated as the particular senses are individually examined. This part of the subject will be considered in another article.

*London; November* 20, 1819. H.

